# Patterns of Care and Outcomes in Verrucous Carcinoma of the Larynx Treated in the Modern Era

**DOI:** 10.3389/fonc.2020.01241

**Published:** 2020-08-07

**Authors:** Thejus T. Jayakrishnan, Stephen Abel, Erik Interval, Athanasios Colonias, Rodney E. Wegner

**Affiliations:** ^1^Allegheny Health Network, Department of Internal Medicine, Pittsburgh, PA, United States; ^2^Division of Radiation Oncology, Allegheny Health Network Cancer Institute, Pittsburgh, PA, United States; ^3^Division of Otolaryngology, Allegheny Health Network Cancer Institute, Pittsburgh, PA, United States

**Keywords:** verrucous carcinoma, larynx, radiation therapy, otolaryngology, laryngectomy

## Abstract

**Background:** Verrucous carcinoma of the larynx (VCL) is a rare form of laryngeal squamous cell carcinoma. We analyzed the National Cancer Database (NCDB) to examine national treatment pattern, identify factors associated with primary radiation therapy (RT), and compare outcomes in patients with Tis-T2 N0 VCL treated primary surgery and primary RT.

**Methods:** We accessed the NCDB from 2004 to 2015 for patients with Tis-T2 N0 VCL and recorded the treatment modality employed. Multivariable logistic regression was used to identify predictors for radiation therapy. Cox regression was used to calculate hazard ratios for survival. A propensity score matched Kaplan–Meier analysis compared primary surgical treatment to definitive radiation.

**Results:** We identified 732 patients with laryngeal verrucous carcinoma from the NCDB. The majority were cTis-T2 (87%) N0 (96%). We identified 286 vs. 110 Tis-T2N0 patients treated primary surgery and with definitive radiation, respectively, for the purpose of this study. Predictors of radiation were treatment at a community center, no insurance, and higher T stage. Cox regression identified increased age, higher comorbidity score, and government insurance as predictive of worse survival. Propensity matching revealed a trend toward worse survival with definitive radiation, with a median survival of 98 months compared to 143 months (*p* = 0.02). When including only T1-2 lesions, that is, invasive disease, the trend toward increased survival with surgery [98 months vs. 135 months (*p* = 0.08)] persisted.

**Conclusion:** The results of the present study support the use of surgery in the management of Tis-T2 N0 VCL when organ preservation is possible.

## Introduction

Verrucous carcinoma of larynx (VCL) is a rare type of squamous cell carcinoma that comprises 1–4% of all primary laryngeal squamous cell cancers (1, 2). It is often seen in older adults, and despite the characteristic papillomatous appearance, association with human papillomavirus is not well-established (2). The management of early-stage laryngeal cancer ranges from surgery alone, radiation therapy alone, or some combination thereof. In contrast, for patients with VCL, the traditional recommendation has been to avoid radiotherapy—primarily due to concern for anaplastic transformation (3, 4). More recently, studies have called into question the concept of radiation-induced anaplastic transformation; nevertheless, radiation therapy continues to be a less preferred treatment approach in management of VCL as contemporary studies continue to demonstrate superior outcomes with surgery (3, 5, 6).

Unfortunately, high powered studies and/or randomized clinical trials on VCL are lacking due to the rarity of this disease entity. As such, guidelines and recommendations regarding the optimal treatment approach are also limited. Given the rarity of VCL and the lack of evidence-based recommendations regarding management, we analyzed the National Cancer Database (NCDB) to examine national treatment patterns, identify factors associated with primary radiation therapy (RT), and compare outcomes in patients with Tis-T2 N0 VCL treated primary surgery and primary RT.

## Methods

**Figure 1:**
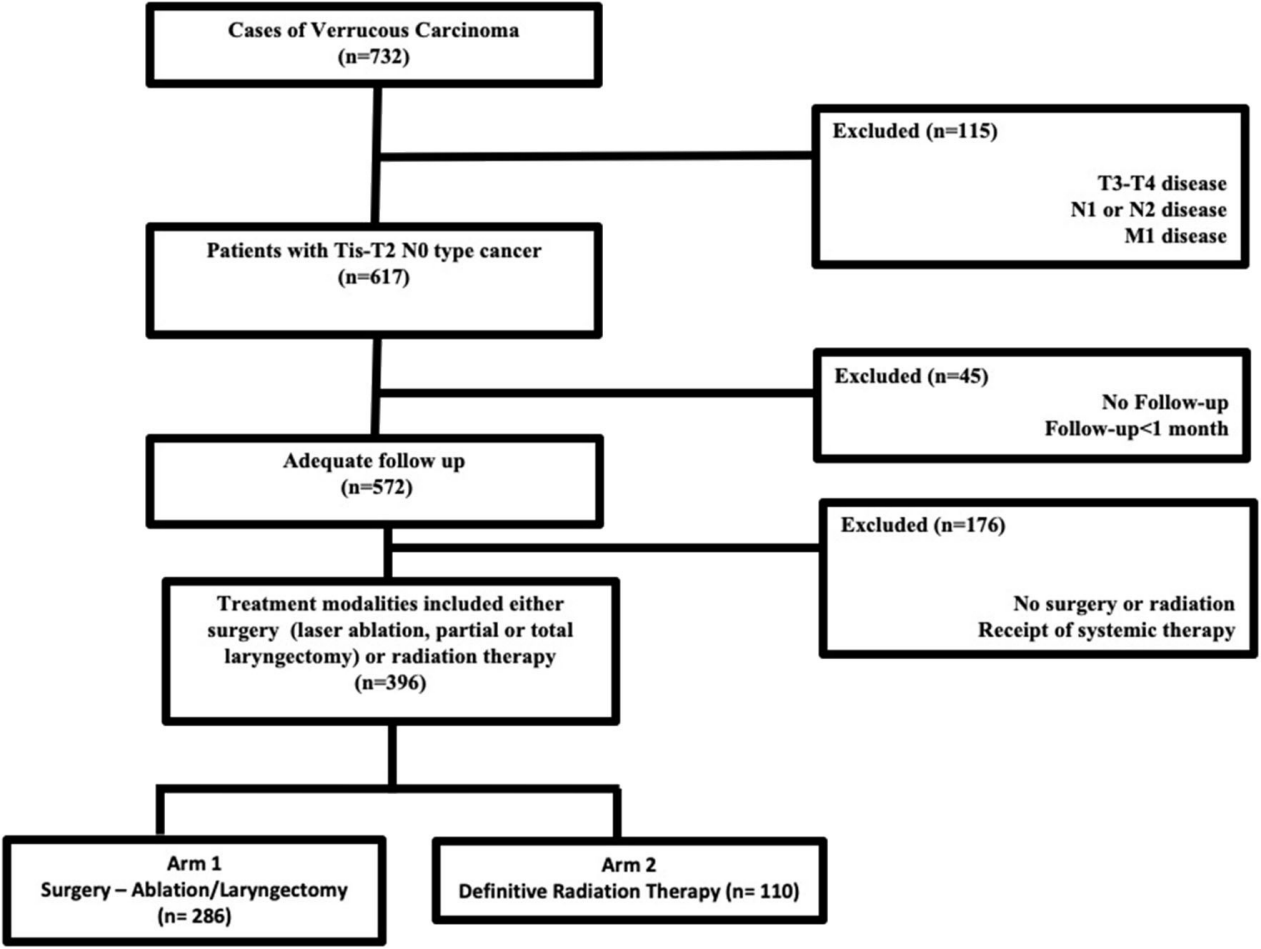
CONSORT diagram outlining the selection criteria for study eligibility.

Jointly maintained by the American Cancer Society and the American College of Surgeons, the NCDB encompasses ~70% of newly diagnosed malignancies each year across the United States. The methods for performing an analysis of the NCDB have been described previously (7, 8). Since the NCDB contains de-identified patient data, this retrospective hospital-based analysis was exempt from Institutional Review Board oversight and ethics approval. We accessed the NCDB for patients diagnosed between 2004 and 2015 with verrucous carcinoma of the larynx (ICD-0-3 code C32, Histology code 8051/3). In the NCDB, databased cases are coded using the American Joint Commission on Cancer (AJCC) cancer staging manual edition in use during the year in which the case was diagnosed. There is no standard mechanism to recode AJCC stage across different editions. We excluded patients with TNM stages other than Tis-T2N0M0, those who were not treated with surgery (ablation/stripping/partial or total laryngectomy) or definitive radiation therapy. According to the NCDB site-specific surgical codes, these include partial excision of the primary site and subtotal/partial laryngectomy or hemilaryngectomy, and include vertical laryngectomy, anterior commissure laryngectomy, and supraglottic laryngectomy. Patients with <1-month follow-up were excluded to account for immortal time bias that is excluding the time period when the outcome could not have occurred and the subject could not have experienced the exposure. Figure 1 outlines the patient selection process. The resultant patient group was split based on treatment modality employed (primary surgery vs. primary radiation therapy) and compared in the analysis.

Race was divided into three broad categories including Caucasian, African-American, or other. Comorbidity was quantified using the widely accepted and verified Charlson/Deyo comorbidity index (9). Socioeconomic data in the patients' residence census tract were provided as quartiles of the percentage of persons with less than a high school education and median household income. The facility type was assigned according to the Commission on Cancer accreditation category. Locations were assigned based on data provided by the US Department of Agriculture Economic Research Service. Insurance status is documented in the NCDB as it appears on the admission page. The American College of Surgeons and the Commission on Cancer have not verified and are not responsible for the analytic or statistical methodology employed or the conclusions drawn from these data by the investigator.

Data were analyzed using Medcalc Version 18 (Ostend, Belgium). Summary statistics are presented for discrete variables. Chi-squared testing compared patient, treatment, and disease-related characteristics between the two treatment groups. Overall survival was calculated in months from time of diagnosis to date of last contact or death as is standard within the NCDB. Kaplan–Meier curves were used to calculate cumulative probability of survival. Log-rank statistics were used to test for significant differences in the cumulative proportions across groups. A Cox proportional hazards model was used for multivariable survival analysis. Factors significant on univariable Cox regression were entered using a stepwise elimination process. Adjusted hazard ratios (HRs) and 95% confidence intervals (95% CIs) are reported using an alpha level of 0.05 to indicate statistical significance.

Propensity score-adjusted survival analysis was used to account for indication bias due to lack of randomization between patients receiving and not receiving radiotherapy (10). Multivariable logistic regression was used to calculate a propensity score indicative of the conditional probability of radiation therapy receipt. The propensity model included observable variables associated with treatment selection on multivariable logistic regression. A Kaplan–Meier analysis was then conducted after matching cases based on the propensity score.

## Results

The dataset included 124,566 patients with cancer of the larynx, of which 732 cases of laryngeal verrucous carcinoma were identified after exclusion of patients with non-verrucous histology. The majority were early stage [i.e., cTis-T2 (87%) N0 (96%)]. Figure 1 outlines the selection process for the cohorts eligible for analyses. Among patients with Tis-T2 N0 M0 cancer who were eligible for analysis, we identified 286 patients (47%) who were treated surgically (ablation/stripping/laryngectomy) vs. 110 (17%) patients who received definitive radiotherapy. Of those undergoing surgery, only 13 (3%) underwent total laryngectomy.

Table 1 displays patient characteristics associated with the cohort eligible for analysis (Tis-T2 N0 M0 or early-stage cancer). This included 93 (23%) AJCC Stage TisN0M0 patients, 217 (55%) T1N0M0, and 86 (22%) T2N0M0. Of the 286 patients who underwent surgery, 76% underwent stripping or ablation while the rest underwent partial or total laryngectomy. Among patients treated with radiation therapy, 87% received 3D therapy and the rest received intensity modulated radiation therapy (IMRT). Median total dose was 66 Gy with conventional fractionation. Median follow-up for the entire cohort was 60 (IQR 27–93) months. It was 62 (IQR 27–94) months for the surgery group vs. 55 (21–89) months for the radiation therapy group.

**Table 1:** Clinicopathologic and treatment characteristics of patients included in the analysis (*n* = 396).

**Characteristics**	**No. (%) or Median (Interquartile Range IQR)**
**Sex**	
Male	354 (89)
Female	42 (11)
**Race**	
White	363 (91)
African American	23 (6)
Other	10 (3)
**Age**	
≤ 60	188 (47)
>60	208 (54)
**Comorbidity score**	
0	278 (70)
1	74 (19)
≥2	44 (11)
**Insurance**	
Not insured	12 (3)
Private payer	165 (41)
Government	204 (52)
Unrecorded	15 (4)
**Education%**	
≥29	98 (25)
20–28.9	117 (30)
14–19.9	107 (27)
<14	71 (18)
**Treatment facility type**	
Community cancer program	40 (10)
Comprehensive community cancer program	137 (35)
Academic/research program Unrecorded	212 (54)
**Treatment facility location**	
Metro	292 (74)
Urban	87 (22)
Rural	8 (2)
Unrecorded	9 (2)
**Income, US dollars**	
<30,000 <30,000–35,000 <35,000–45,999	92 (23) 98 (25) 83 (21)
>46,000	119 (31)
**Distance to treatment facility, miles**	
≤ 13 miles >13 miles	194 (49) 201 (51)
**Year of diagnosis**	
2004–2006	126 (32)
2007–2009	105 (27)
2010–2012	90 (23)
2013–2016	75 (19)
**AJCC stage**	
TisN0M0	93 (23)
T1N0M0	217 (55)
T2N0M0	86 (22)
**Grade**	
Well-differentiated Moderately differentiated Poorly differentiated Not recorded	153 (39) 16 (4) 1 (0.3) 226 (57)
**Treatment characteristics**	
Surgery type	286
Stripping or ablation	216 (76)
Partial or total laryngectomy	70 (24)
Type of radiation therapy 3D therapy Intensity modulated radiation therapy (IMRT)	110 96 (87%) 14 (13%)
Total doses of radiation therapy (median, IQR) Fractionation of radiation therapy (median, IQR)	66 (63–70) Gy 33 (23–35)Fractions
Follow-up overall	60 (27–93) months
Follow-up for surgery group	62 (27–94) months
Follow-up for radiation therapy group	55 (21–89) months

**Figure 2:**
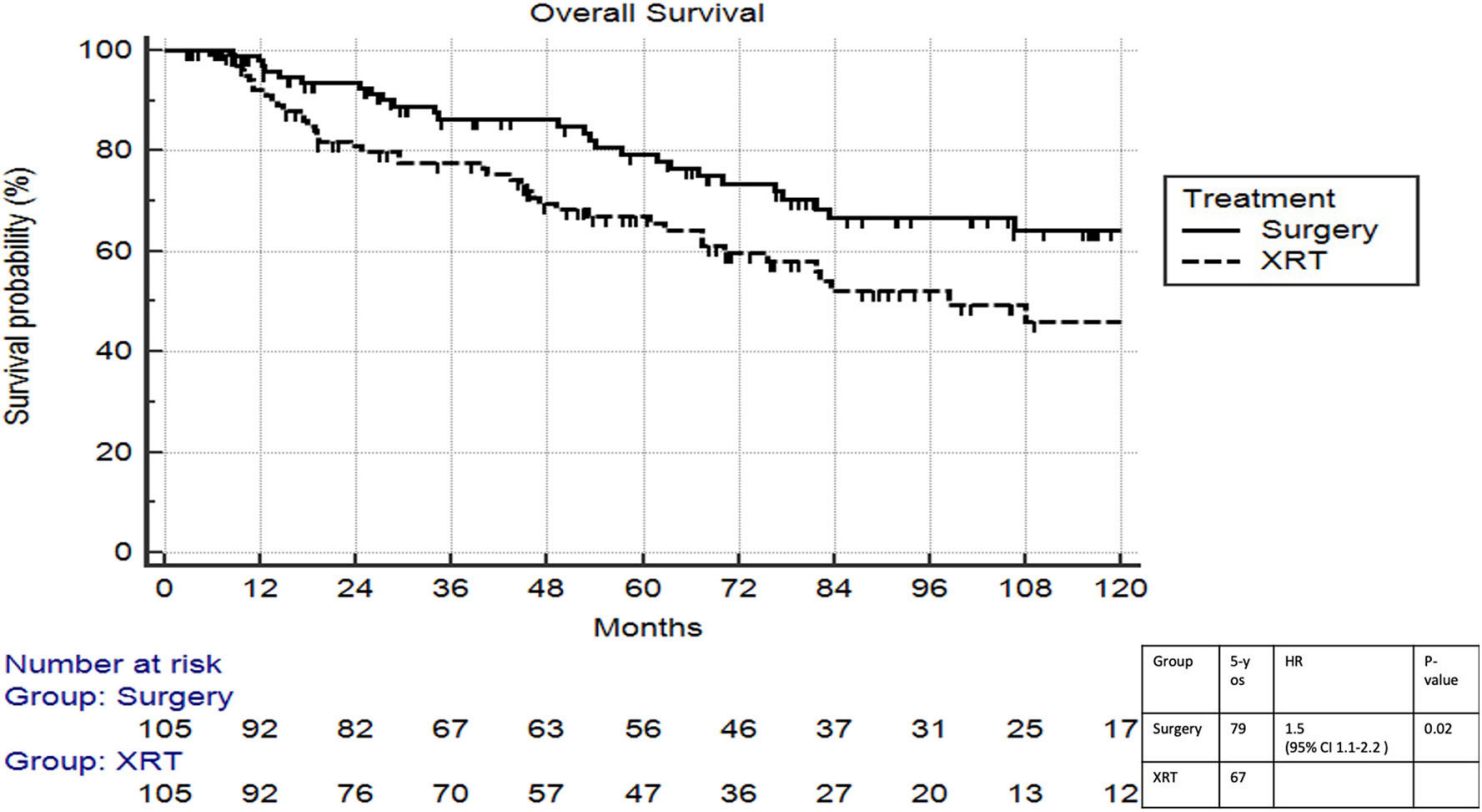
Propensity matched survival analysis of radiation therapy vs. surgery in Tis-T2 N0 verrucous carcinoma of the larynx.

**Figure 3:**
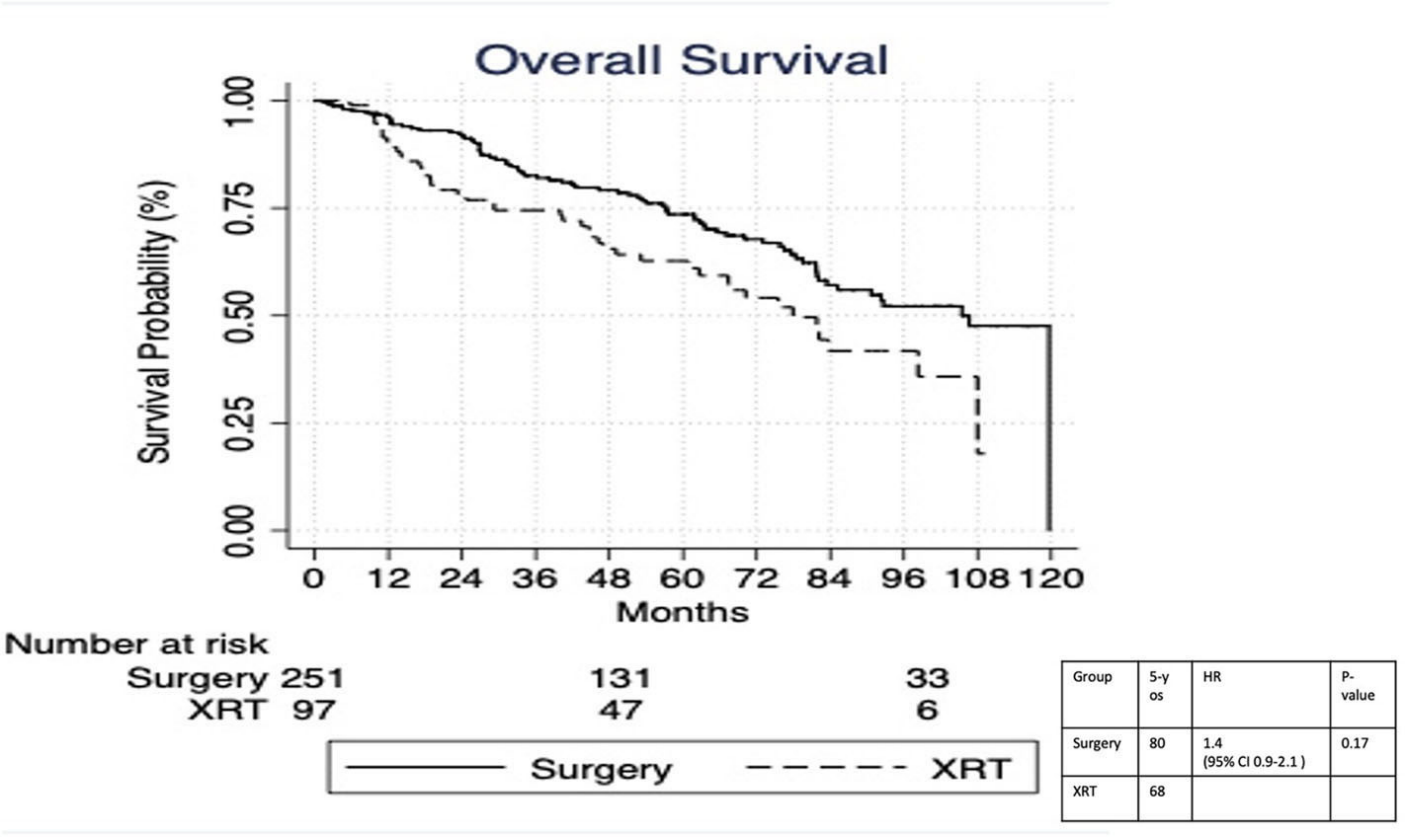
Survival analysis of radiation therapy vs. surgery in Tis-T2 N0 verrucous carcinoma of the larynx prior to propensity matching.

On multivariable logistic regression, predictors of radiation therapy receipt among early-stage patients (Tis-T2 N0 M0) included treatment at a community center, no insurance, and higher T stage (Table 2). Negative prognostic covariates identified on multivariate Cox regression included increased age, higher comorbidity score, and government insurance (Table 3). Propensity matching resulted in 105 matched pairs, and propensity-matched Kaplan–Meier analysis revealed worse survival with definitive radiation (Figure 2). The median overall survival was 143 (95% CI 121–159) months vs. 98 (95% CI 70–139) months, with *p* = 0.0216 for surgery compared to radiation therapy (Figure 2). For reference, the survival curves prior to propensity matching are shown in Figure 3. In cases of invasive disease (i.e., T1–T2), this survival trend persisted (135 months vs. 98 months, *p* = 0.0805) in favor of surgery, although not statistically significant. The 5-year overall survival for the groups was 79% for surgery vs. 67% radiation therapy—HR 1.54 (95% CI 1.07–2.2, *p* = 0.021). Disease-specific survival could not be calculated as data regarding disease recurrence are not available through the NCDB.

**Table 2:** Multivariate logistic regression for likelihood of receiving definitive radiation therapy for Tis-T2 N0.

**Characteristic (*n* = 110)**	**Odds ratio (95% CI)**	***p***
**Age**		
≤ 60	Reference	
>60	1.85 (0.95–3.59)	0.0703
**Comorbidity score**		
0	Reference	
1	1.16 (0.59–2.26)	0.6634
≥2	0.81 (0.33–1.96)	0.6390
**Distance**		
≤ 13 miles	Reference	
>13 miles	0.26 (0.14–0.50)	<0.0001
**Facility type**		
Community cancer center	Reference	
Comprehensive community cancer center	0.65 (0.27–1.57)	0.3406
Academic/research program	0.32 (0.13–0.77)	0.0106
**Grade**		
Well-differentiated	Reference	
Moderately differentiated	0.53 (0.15–1.90)	0.3285
Poorly differentiated	6.93 (0.28–17.34)	0.2383
Education, % without high school diploma		
≥29	Reference	
20–28.9	0.67 (0.31–1.46)	0.3176
14–19.9	0.66 (0.27–1.60)	0.3568
<14	1.25 (0.46–3.40)	0.6564
**Income, USD**		
<30,000	Reference	
30,000–34,999	0.93 (0.40–2.14)	0.8616
35,000–45,999	0.61 (0.24–1.56)	0.3050
≥46,000	0.91 (0.34–2.45)	0.8591
**Insurance**		
None	Reference	
Private	0.25 (0.06–0.99)	0.0477
Government	0.38 (0.09–1.54)	0.1756
**Location**		
Metropolitan	Reference	
Urban	1.23 (0.58–2.62)	0.5926
Rural	3.91 (0.58–26.37)	0.1611
**Race**		
Caucasian	Reference	
African American	0.75 (0.25–2.22)	0.5999
Other	0.55 (0.09–3.45)	0.5200
**Gender**		
Male	Reference	
Female	0.79 (0.33–1.86)	0.5845
**Stage**		
Tis	Reference	
T1	5.98 (2.40–14.94)	0.0001
T2	15.82 (5.70–43.93)	<0.0001
**Year**		
2004–2006	Reference	
2007–2009	0.74 (0.36–1.49)	0.3943
2010–2012	0.72 (0.35–1.49)	0.3718
2012–2015	0.56 (0.25–1.23)	0.1498

*Propensity score calculated with distance, facility, T stage, and insurance*.

**Table 3:** Propensity matched multivariate Cox regression analysis for overall survival (*n* = 105 matched pairs).

**Characteristic**	**HR (95% CI)**	***p***
**Age**		
≤ 60	Reference	
>60	1.75 (1.09–2.83)	0.0214
**Comorbidity score**		
0	Reference	
1	1.15 (0.74–1.76)	0.5371
≥2	2.04 (1.26–3.31)	0.0038
**Insurance**		
None	Reference	
Private		
Governmental	3.93 (2.37–6.55)	<0.0001

## Discussion

Although some early studies recommended close monitoring and non-aggressive management of VCL, definitive treatment is now always recommended either by definitive surgery or definitive radiation therapy or a combination thereof with chemotherapy reserved for more advanced stages (1, 11). In the current study looking at the Tis-T2 N0 M0 VCL, we identified several predictors of radiotherapy including treatment at a community center, no insurance, and T1–T2 tumor compared to Tis cancer. In this first propensity matched analysis of the two modalities of treatment of Tis-T2 N0 M0 VCL, we demonstrated superior survival outcomes for patients undergoing surgery with advanced age, presence of comorbidities, and lack of private insurance as predictors for poor outcome.

The identified differences in management may reflect differences in practice patterns such as radiation therapy being administered more commonly in community practice (12). The increased usage of radiation therapy, a strategy that is historically not recommended in this condition among patients with no insurance and therefore a vulnerable group, is concerning. The relationships between type of treatment and sociodemographic factors have been demonstrated previously, but it is difficult to ascertain the mechanisms based on the data available from the NCDB (12). It is also important to consider that insurance history is not a static factor, and therefore, further determination of this association would need prospective studies (13).

Historically, surgery has been the favored approach due to reports of anaplastic transformation and secondary metastasis associated with radiotherapy (4). While the improved outcomes with definitive surgery (5-year overall survival 87%) have been reported previously, we also noticed poorer outcomes in those treated with a combination of surgery and radiation (1, 3, 11). This is in contrast to other variants of laryngeal cancers, and the current study is limited in identifying the underlying mechanisms (14). Earlier studies have attributed this to anaplastic transformation of the cancer (rates as high as 11%) resulting in adverse outcomes (4). While subsequent studies failed to reproduce comparable results, they did demonstrate favorable surgical outcomes compared to patients treated with radiotherapy (3, 5, 6, 14–16).

The above studies suffer from obvious limitations including selection bias and low statistical power considering their retrospective nature and small patient volume (i.e., small retrospective series or case reports). In addition, these studies were predominantly published before 2000. There have been significant advancements in radiation therapy since the publication of these studies, and while conservative surgery is possible in most cases, 20% or more patients may not be eligible for larynx preservation surgery (17). Even in earlier studies, it has been demonstrated that local control with radiation therapy for VCL (5-year rate of local control−59%), while inferior to other types of laryngeal cancer, offers similar overall survival following salvage surgical resection (18). As such, less invasive treatment strategies such as radiotherapy have been suggested as potential alternatives to surgical resection (3, 15). This recommendation cannot be made based on the findings from the present study.

As discussed previously, the existing literature on radiation for VCL suffers from significant bias as they were essentially all retrospective studies with heterogeneity in the groups used for comparison (17). It is interesting that despite the propensity matching technique in the current study, patients with VCL achieved better survival with surgery compared to radiotherapy as radiotherapy patients should have at least theoretically had the potential for surgical salvage in the setting of local failure. Patients who failed following surgery can receive radiation therapy as salvage also. These effects could not be well-evaluated as the NCDB lacks data on local failure. While the previous population-based studies have found similar outcomes, selection bias among high-stage lesions was suggested as a limitation (6). We attempted to minimize this through propensity score matching and excluding advanced stage presentations. But it should be noted that patients not amenable to matching are excluded during propensity matching with implications on representativeness of the matched cohorts with regard to the true population (19). Thus, it should be assumed that unidentified biases still exist that could be uncovered only through randomized controlled trials. It should also be noted that although survival analysis favored surgery overall, it was not statistically significant in the T1–T2 group and could be because the study was not powered to detect the differences. Certainly, surgery may be truly superior to radiation therapy due to hitherto unknown biological factors related to this rare cancer. As is typical with these types of analyses, this study was limited by the data provided in the NCDB due to its retrospective nature. Compounding this, the NCDB lacks information on adverse events, local failure, and quality of life, all of which play an important role in management and ultimately outcome.

## Conclusions

The results of the present study support the use of surgery in the management of Tis-T2 N0 VCL when organ preservation is possible. Radiation therapy should be reserved for poor surgical candidates or in circumstances where acceptable functional outcomes cannot be achieved with surgery.

## Data Availability Statement

The datasets generated for this study are available on request to the corresponding author.

## Author Contributions

RW, TJ, and SA conceived the project and performed the analysis. RW supervised the project. TJ, SA, EI, AC, and RW contributed to the writing, editing, and production of the final version of the manuscript. All authors contributed to the article and approved the submitted version.

## Conflict of Interest

The authors declare that the research was conducted in the absence of any commercial or financial relationships that could be construed as a potential conflict of interest.
